# Clinical and epidemiological features of Lyme neuroborreliosis in adults and factors associated with polyradiculitis, facial palsy and encephalitis or myelitis

**DOI:** 10.1038/s41598-023-47312-4

**Published:** 2023-11-14

**Authors:** Daiva Radzišauskienė, Jurgita Urbonienė, Arminas Jasionis, Aušra Klimašauskienė, Radvilė Malickaitė, Agnė Petrulionienė, Monika Vitkauskaitė, Gintaras Kaubrys

**Affiliations:** 1 Clinic of Infectious Diseases and Dermatovenerology, Institute of Clinical Medicine, Faculty of Medicine, Vilnius University, Vilnius, Lithuania; 2 Center of Infectious Diseases, Faculty of Medicine, Vilnius University, Vilnius, Lithuania; 3 Clinic of Neurology and Neurosurgery, Institute of Clinical Medicine, Faculty of Medicine, Vilnius University, Vilnius, Lithuania; 4 Clinic of Cardiac and Vascular Diseases, Institute of Clinical Medicine, Faculty of Medicine, Vilnius University, Vilnius, Lithuania; 5 Constitution Clinic, Vilnius, Lithuania

**Keywords:** Infectious diseases, Neurological manifestations

## Abstract

The clinical course of Lyme neuroborreliosis (LNB) is highly variable. Delayed diagnosis and treatment still remain actual challenges. Moreover, there is a lack of studies analyzing the factors associated with different LNB syndromes. We aimed to analyze clinical and epidemiological features of LNB in hospitalized adults in eastern Lithuania. A retrospective study was performed for patients presenting in the years 2010–2021. A total of 103 patients were included in the study, 100 with early, and three with late LNB. Patients with early LNB most often presented polyradiculitis [75/100, (75%)], which was also the most common initial neurological syndrome. Peripheral facial palsy was diagnosed in 53/100 (53%) patients, in 16/53 (30.2%) cases both facial nerves were affected. Encephalitis or myelitis was diagnosed in 14% of patients with LNB. A total of 76/103 (73.8%) patients were discharged with residual symptoms or signs. One patient presenting encephalomyelitis died because of bacterial complications. The absence of observed erythema migrans (EM) was the predictor of peripheral facial palsy, while female sex and EM untreated with antibiotics were predictors of isolated polyradiculitis. A fever of ≥ 38 ° °C and pleocytosis of ≥ 300 × 10^6^/l were associated with the development of encephalitis or myelitis in patients with early LNB.

## Introduction

Lyme borreliosis (LB) is the most common tick-borne disease in Europe, including Lithuania. In Europe, 65,500 people are affected by this disease annually^1^. The incidence is the highest in Scandinavian and the Baltic states in northern Europe, and in Austria, Czechia, Germany, and Slovenia in central Europe^2^. In Lithuania, a significant rise of morbidity was observed in the years 2010–2021, and incidence rate varied between 63.7 and 117.8 cases per 100,000 inhabitants^3^.

LB is a zoonosis transmitted by ticks and caused by *Borrelia (B.) burgdorferi *sensu lato (s.l.) spirochete complex^4,5^. *B. burgdorferi* s.l*.* is classified into 3 main genospecies: *B. burgdorferi *sensu stricto (s.s.), *B. afzelii, B. garinii.*

LB can be a multisystem, multistage disease but does not always progress beyond the first stage. Erythema migrans (EM) is the most common manifestation of LB. Early Lyme neuroborreliosis (LNB) is the second most frequent clinical manifestation in Europe, which occurs in 10–15% of all LB cases^6^. The clinical course of LNB is highly variable. Any part of the nervous system may be involved, but the disease usually manifests as lymphocytic meningitis, meningopolyradiculitis, and/or cranial neuritis^5^. Encephalitis and myelitis are the most severe forms but occur rarely^5^. It is important to diagnose this infectious disease as early as possible in order to introduce appropriate treatment before it results in axonal loss^7^ or other neurological deficits that impair the patients’ quality of life and requires long rehabilitation. Although the clinical manifestation of LNB is relatively well studied, factors associated with various LNB syndromes [e.g. age, sex, co-morbidities, manifestation of EM, fever, inflammatory changes in cerebrospinal fluid (CSF)] are still insufficiently analyzed; delayed diagnosis and treatment remain actual challenges as well.

The aim of this study was to analyze clinical and epidemiological features of LNB in hospitalized adults, and to identify the factors associated with the development of polyradiculitis, peripheral facial palsy, and encephalitis or myelitis.

## Results

### Epidemiological and demographic data

The study included 103 patients. A total of 95/103 (92.2%) patients were diagnosed with definite LNB (DLNB). The possible LNB (PLNB) was diagnosed in 8/103 (7.8%) patients, and 5 of them had the presence of EM during hospitalization (Table 1). A total of 34/103 patients (33%) had presence or reliable history of recent EM within 120 days before the onset of the neurological symptoms or signs, and 31/34 (91%) of these cases were untreated with antibiotics. Three patients (3/34) noticed EM when they developed neurological symptoms or signs.

**Table 1:** Baseline data and initial neurological symptoms and signs of patients with Lyme neuroborreliosis.

Parameters	Value
Age, years, median (IQR); min–max, N = 103	60 (46–70); 18–90;
Sex, male, N (%)	50 (48.5)
History of tick bite ≤ 120 days before LNB symptoms, N (%)	40 (38.8)
Pre-existing conditions, N (%)	56 (54.4)
Arterial hypertension	41 (39.8)
Diabetes	7 (6.8)
Incubation period (days), median (IQR); min–max, N = 27	35 (27–81); 10–113
Time from the onset of EM until the first symptom of LNB (days), median (IQR); min–max, N = 24	18 (14–36.3); 4–98
Time from the onset of LNB symptoms until hospitalization (days), median (IQR); min–max, N = 95	20 (8–31); 0–257
Defined LNB, N (%)	95 (92.2)
Possible LNB, N (%)	8 (7.8)
The presence of EM, N (%)	5/8 (62.5)
Isolated polyradiculitis	2
The presence of EM	2/2
Polyradiculitis + peripheral facial palsy	2
The presence of EM	½
Isolated peripheral facial palsy*	2
The presence of EM	0
Only lymphocytic meningitis	2
The presence of EM	2/2
The initial neurological symptoms and signs of early LNB, N (%)	
The radicular pain	71 (71)
Peripheral facial palsy	14 (14)
Signs of encephalitis	10 (10)
Occipital neuralgia	6 (6)

*Concomitant with CSF pleocytosis, LNB Lyme neuroborreliosis, EM erythema migrans.

The most common co-morbidities were arterial hypertension (39.8%) and diabetes (6.8%). Other concomitant diseases were cancer (3.8%), coronary heart disease (3.8%), and chronic kidney disease (2.9%).

**Figure 1:**
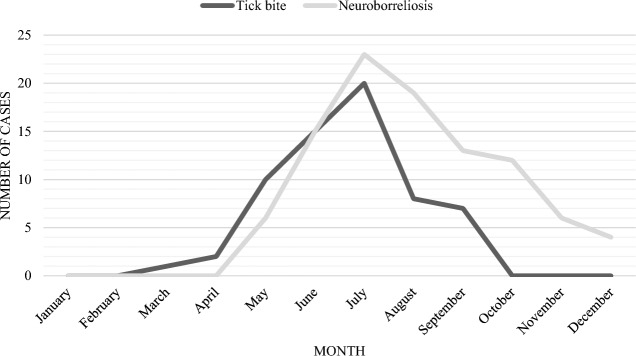
The seasonal distribution of reported tick bites and Lyme neuroborreliosis. *Note*: tick bite curve shows data of patients who reported only a single tick bite (n = 63). A total of 5 patients did not report when LNB symptoms began, therefore n = 98.

The highest number of LNB cases occurred in July (Fig. 1). Most often, patients became infected in their living area—the city of Vilnius (46/103 patients, 44.7%) and the Vilnius region (16/103 patients, 15.5%). Other areas where patients became infected were: the city and region of Trakai (8/103 (7.8%) of cases), the region of Molėtai [4/103 (3.9%)], the city and region of Širvintos, the region of Švenčionys, the region of Panevėžys (3/103 (2.9%) of cases in each). In the regions of Šalčininkai, Ukmergė, Utena, Zarasai, 2 patients each (1.9%) became infected. One patient each (0.97%) became infected in the cities of Varėna, Visaginas, Druskininkai, Birštonas and in the regions of Elektrėnai and Kaunas. The areas of becoming infected were unknown in 6/103 (5.8%) patients.

### Clinical presentation

**Figure 2:**
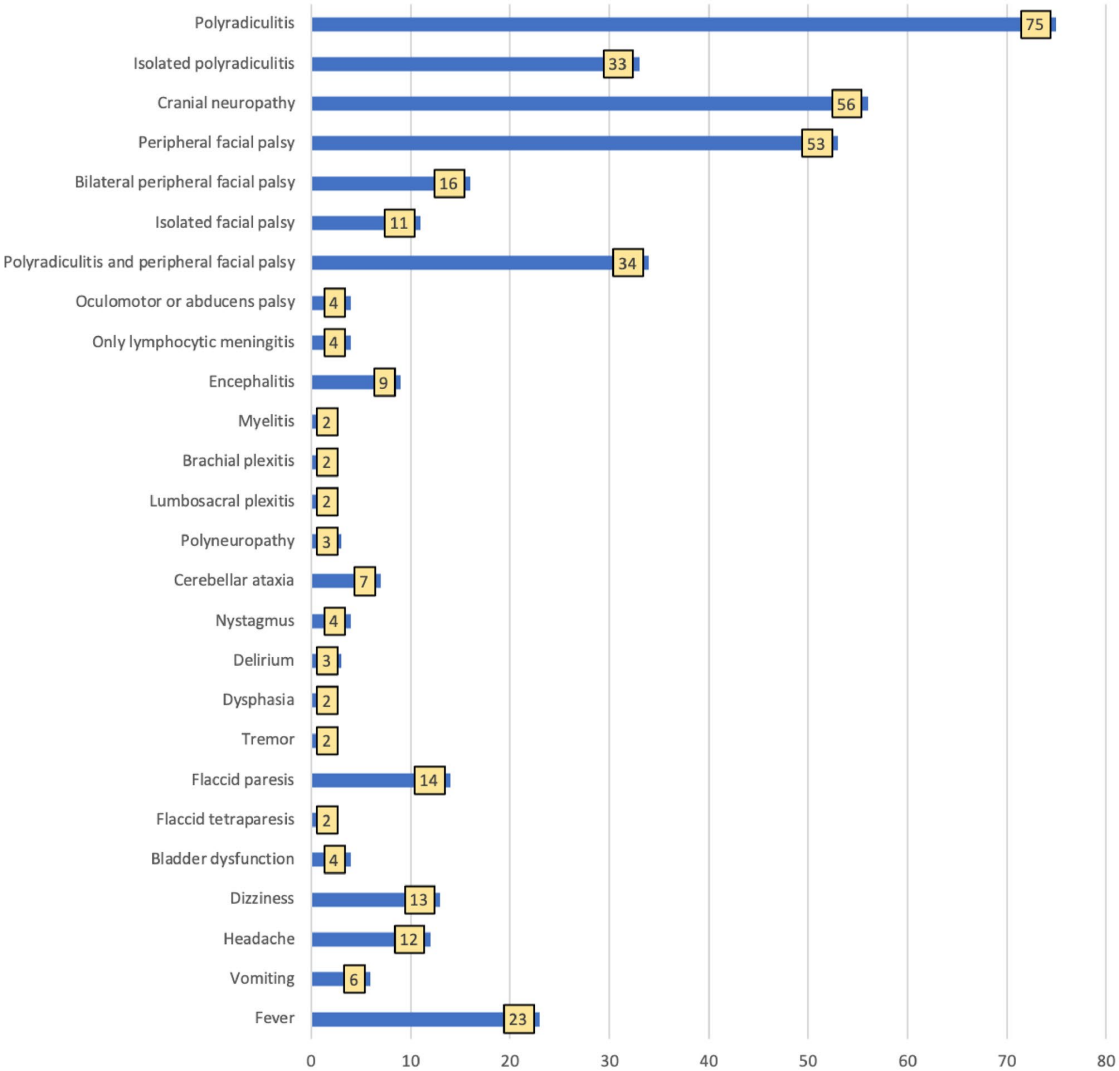
The clinical presentation of early Lyme neuroborreliosis, N = 100.

The majority of patients [100/103, (97.1%)] were diagnosed with early LNB. Patients with early LNB most often presented polyradiculitis [75/100 cases, (75%)] (Fig. 2). The radicular pain was the most frequent first neurological symptom (Table 1). The most common location of radicular pain was lower back [36/75 cases, (48%)], followed by shoulder girdle [22/75 cases, (29.3%)]. Legs and chest were affected in 20/75 cases each (26.7%), arms in 7/75 cases (9.3%), neck in 14/75 cases (18.7%). A total of 23/75 (30.7%) patients with polyradiculitis had the presence or reliable history of recent EM, while 22 cases were untreated with antibiotics.

Cranial neuropathies were diagnosed in 56/100 cases (56%) (Fig. 2) and were reported less often in patients with EM compared to patients without the history of EM ((13/34 (38.2%) vs 43/66 (65.2%), *p* = 0.010).

Peripheral facial palsy was diagnosed in 53/100 patients (53%) (Fig. 2); in 16/53 cases (30.2%) both facial nerves were affected. The median time from unilateral to bilateral peripheral facial palsy was 3 days (IQR 0–5, min 0, max 10, n = 15).

Nine patients (9%) presented symptoms and signs of encephalitis (Fig. 2). Ataxia was the most prevalent encephalitis symptom, appearing in seven cases; in one case, it was so severe that the patient was unable to walk for a month. Memory disorders, facial myoclonus, writing problems, and dysphagia were all reported with frequency of 1/100 cases (1%) each. A total of 7/9 patients had a fever median 38.5 °C ((37.8–39); min 37.4 °C–max 39 °C). Two patients had a two-wave fever.

Myelitis was diagnosed in two patients with early LNB. A 22-year-old patient presented spastic paraparesis of legs, sensory impairment, urinary retention, and left peripheral facial palsy. The patient had no signs of encephalitis and no abnormalities in head magnetic resonance imaging (MRI). The patient had a history of untreated EM within 14 days prior to the onset of neurologic symptoms. At the beginning of the disease, the intrathecal synthesis of *B. burgdorferi* s.l. antibodies, as well as *B. burgdorferi* s.l. antibodies in the serum were not detected, but inflammatory changes were found in the CSF. After 2 weeks, the intrathecal synthesis of *B. burgdorferi* s.l. IgM was detected, but antibodies were not detected in serum. *B. burgdorferi* s.l. IgM antibodies were detected 3 weeks after the onset of the illness. An 88-year-old patient presented fever and paresis of left leg. After 10 days, the patient developed stupor, lower paraparesis, accompanied by sensory loss and urinary retention. The patient was previously healthy and active, had no history of EM and tick bite, but often visited forests. Inflammatory changes were found in the CSF, as well as the intrathecal synthesis of *B. burgdorferi* s. l. IgG was proven by the pathological CSF/serum antibody index (AI). Spinal MRI showed a transverse T2 hyperintense lesion at the Th1-3 level (Table A1 online). Other possible diseases listed in the methods section were excluded.

Univariable logistic regression showed that female sex and EM untreated with antibiotic were associated with increased odds ratio of having isolated polyradiculitis in patients with early LNB. In multivariable regression, female sex and EM untreated with antibiotics remained statistically significant predictors of isolated polyradiculitis (Table 2).

**Table 2:** The predictors for developing isolated polyradiculitis, peripheral facial palsy and encephalitis or myelitis in patients with early LNB.

	Univariable OR (95% CI)	Multivariable OR (95% CI)
Predictors for isolated polyradiculitis		
Age (years)	1.02 (1.00–1.05); *p* = 0.125	
Female sex	3.95 (1.59–9.80); *p* = 0.003	3.91 (1.45–10.55); *p* = 0.007
Untreated EM	3.26 (1.34–7.96); *p* = 0.009	3.24 (1.23–8.54); *p* = 0.018
Concomitant diseases	0.97 (0.42–2.25); *p* = 0.949	
Fever ≥ 38 °C	0.20 (0.02–1.66), *p* = 0.137	
Pleocytosis ≥ 300 × 10^6^/l	0.42 (0.13–1.37), *p* = 0.151	
Protein in CSF	0.50 (0.24–0.99), *p* = 0.045	0.65 (0.32–1.32); *p* = 0.230
Predictors for peripheral facial palsy		
Age (years)	0.98 (0.95–1.00); *p* = 0.049	0.98 (0.95–1.00); *p* = 0.088
Male sex	2.27 (1.02–5.07); *p* = 0.045	1.63 (0.66–3.97); *p* = 0.287
Absence of observed EM	2.49 (1.06–5.81); *p* = 0.036	2.84 (1.15–7.00); *p* = 0.024
Concomitant diseases	0.83 (0.38–1.83); *p* = 0.643	
Fever ≥ 38 °C	0.56 (0.15–2.11); *p* = 0.390	
Pleocytosis ≥ 300 × 10^6^/l	0.74 0.28–1.94); *p* = 0.541	
Protein (g/l)	1.14 (0.74–1.78); *p* = 0.555	
Predictors for encephalitis or myelitis		
Age	1.01 (0.97–1.05); *p* = 0.676	
Male sex	2.67 (0.65–10.97); *p* = 0.174	
Untreated EM	0.95 (0.23–3.94); *p* = 0.943	
Concomitant diseases	1.23 (0.33–4.75); *p* = 0.738	
Fever ≥ 38 °C	17.00 (3.67–78.74); p < 0.001	12.11 (1.88–77.78); *p* = 0.009
Pleocytosis ≥ 300 × 10^6^/l	12.5 (2.88–54.25); *p* = 0.001	10.58 (1.67–67.03); *p* = 0.012
Protein (g/l)	2.09 (1.17–3.74); *p* = 0.013	1.00 (0.46–2.14); *p* = 0.989

*EM* erythema migrans, *CSF* cerebrospinal fluid.

Variables with *p* < 0.05 in univariable regression were included into multiple regression models.

Model for isolated polyradiculitis: Hosmer and Lemenshow test Χ^2^ = 16.54, *df* = 8, *p* = 0.035; Nagelkerke R^2^ = 0.245.

Model for facial paresis: Hosmer and Lemenshow test Χ^2^ = 9.13, *df* = 8, *p* = 0.331; Nagelkerke R^2^ = 0.142.

Model for encephalitis or myelitis: Hosmer and Lemenshow test Χ^2^ = 6.64, *df* = 8, *p* = 0.576; Nagelkerke R^2^ = 0.402.

Univariable regression revealed that age, male sex, and the absence of observed EM were associated with increased odds ratio for the development of peripheral facial palsy in patients with early LNB. In multivariable regression, the absence of observed EM remained statistically significant, associated with 2.84 (95% CI 1.15–7.00) fold increased odds ratio for developing facial palsy (Table 2).

A fever of ≥ 38 °C and pleocytosis of ≥ 300 × 10^6^/l predicted encephalitis and/or myelitis in patients with early LNB (Table 2).

Late LNB was diagnosed in 3/103 patients (2.9%). Two of them presented with encephalitis, and one with encephalomyelitis. A 66-year-old patient with encephalitis presented dizziness, cerebellar ataxia, diplopia, and dysphagia. The patient had a history of self-limiting peripheral facial palsy. A 42-year-old patient presented headache, cerebellar ataxia, and diplopia. A 71-year-old patient with encephalomyelitis presented fever, headache, tremor, signs of vestibulocochlear neuropathy, and signs of myelitis.

### Laboratory and instrumental findings

**Figure 3:**
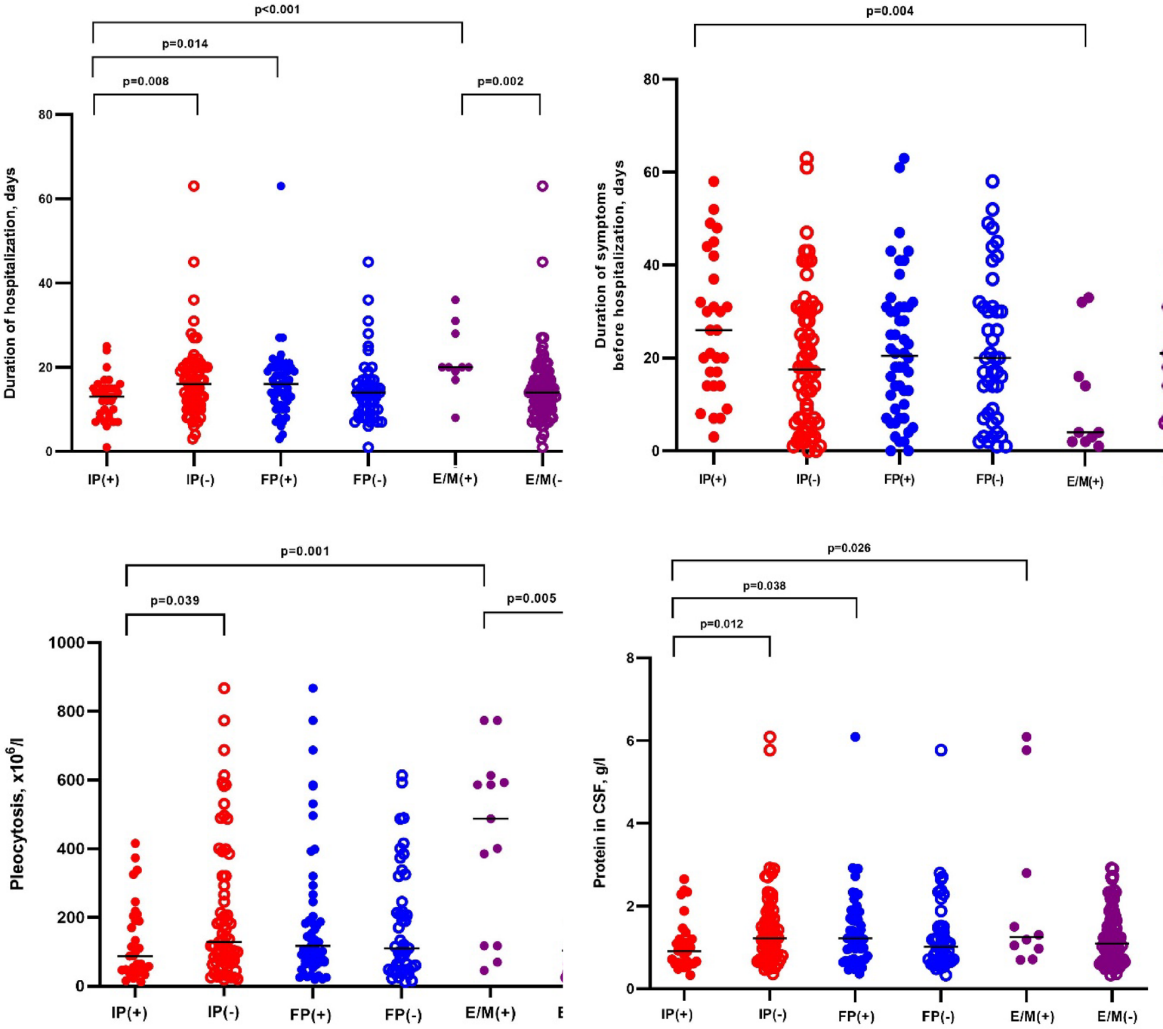
Comparison of duration of symptoms before hospitalization, duration of hospitalization, pleocytosis and protein in cerebrospinal fluid in patients with early Lyme neuroborreliosis. IP(+) early LNB patients with isolated polyradiculitis; IP(−) early LNB patients excluding patients with isolated polyradiculitis; FP(+) early LNB patients with facial palsy; FP(−) early LNB patients without facial palsy; E/M(+) early LNB patients with encephalitis or myelitis; E/M(−) early LNB patients excluding patients with encephalitis or myelitis. *LNB* Lyme neuroborreliosis.

There were no significant abnormalities in hematology tests, C reactive protein (CRP), or erythrocyte sedimentation rate (ESR) (Table 3). CSF analysis was performed in all LNB patients. All patients with early LNB had elevated cell count in CSF (median 112 × 10^6^/l, minimum 12 × 10^6^/l − maximum 867 × 10^6^/l) (Table 4). The intrathecal synthesis of neither IgM, nor IgG *B. burgdorferi* s. l. was detected in eight patients whose CSF was examined less than 8 weeks after the onset of the disease. Higher pleocytosis was observed in patients with encephalitis and/or myelitis (p = 0.005) (Fig. 3).

**Table 3:** Blood analysis of patients with Lyme neuroborreliosis.

Variables	Early LNB	Late LNB
Leukocytes* × 10^9^/l, median (IQR), min–max, n	8.13 (6.35–9.7); 2.86–16; N = 99	55.9; 4.13–10.6; N = 3
CRP, mg/l, median (IQR), min–max, n	3.71 (1.31–5.84); 0.01–26.41; N = 94	2.5; 2–3; N = 3
ESR, mm/h median (IQR), min–max, n	9.5 (6–17); 1–88; N = 85	25; 7–25; N = 3
*B. burgdorferi* s.l. IgM (ELISA) positive, n (%)	79/100 (79)	2 (66.7)
*B. burgdorferi* s.l. IgG (ELISA) positive, n (%)	86/100 (86)	3 (100)
Both positive, n (%)	70/100 (70)	1 (33.3)
Both negative, n (%)	4/100 (4)	0

*The data of one patient with lymphocytic leukemia are excluded.

**Table 4:** Cerebrospinal fluid analysis in patients with Lyme neuroborreliosis.

Variables	Early LNB (all patients)	Cranial neuropathies*	Encephalitis*	Myelitis*	Late LNB
Pleocytosis, cellsx10^6^/l, median (IQR); min–max; N	112 (57.75–238.25); 12–867; N = 100	117 (69–260.75); 19–867; N = 56	400 (93–589); 45–613; N = 9	682.50 (592–0); 592–973; N = 2	230; 135–305; N = 3
Lymphocytes %, median (IQR); min–max; N	97.5 (92–100); (10–100); N = 98	97 (90–100); 10–100; N = 55	98 (80.50–100); 65–100; N = 5	51 (10–92); N = 2	90; 21–97; N = 3
Protein, g/l, median (IQR); min–max; N	1.10 (0.70–1.65); 0.33–6.09	1.23 (0.71–1.69); 0.36–6.09; N = 56	1.3 (0.84–4.29); 0.70–6.09; N = 9	3.41; 1.05–5.77; N = 2	3.3; 1.8–6.47; N = 3
Glucose, mmol/l, median (IQR); min–max; N	3.04 (2.60–3.44); 0.40–5.50	3 (2.58–3.50); 0.4–4.38; N = 55	2.9 (2.21–3.73); 1.60–5.50; N = 9	2.03; 0.4–3.65; N = 2	2.6; 1.88–3.1; N = 3
Intrathecal synthesis of *B. burgdorferi* s.l. N (%)					
IgM	60/95 (63.2)	34/52 (65.4)	4/9 (44.4)	1/2 (50)	1/3 (33.3)
IgG	79/98 (77.6)	45/55 (81.8)	8/9 (88.9)	1/2 (50)	3/3 (100)
Either IgM or IgG, or both	92/100 (92)	52/56 (92.9)	9/9 (100)	2/2 (100)	3/3 (100)

*Patients with early LNB.

*LNB* Lyme neuroborreliosis, *IQR* interquartile range.

**Figure 4:**
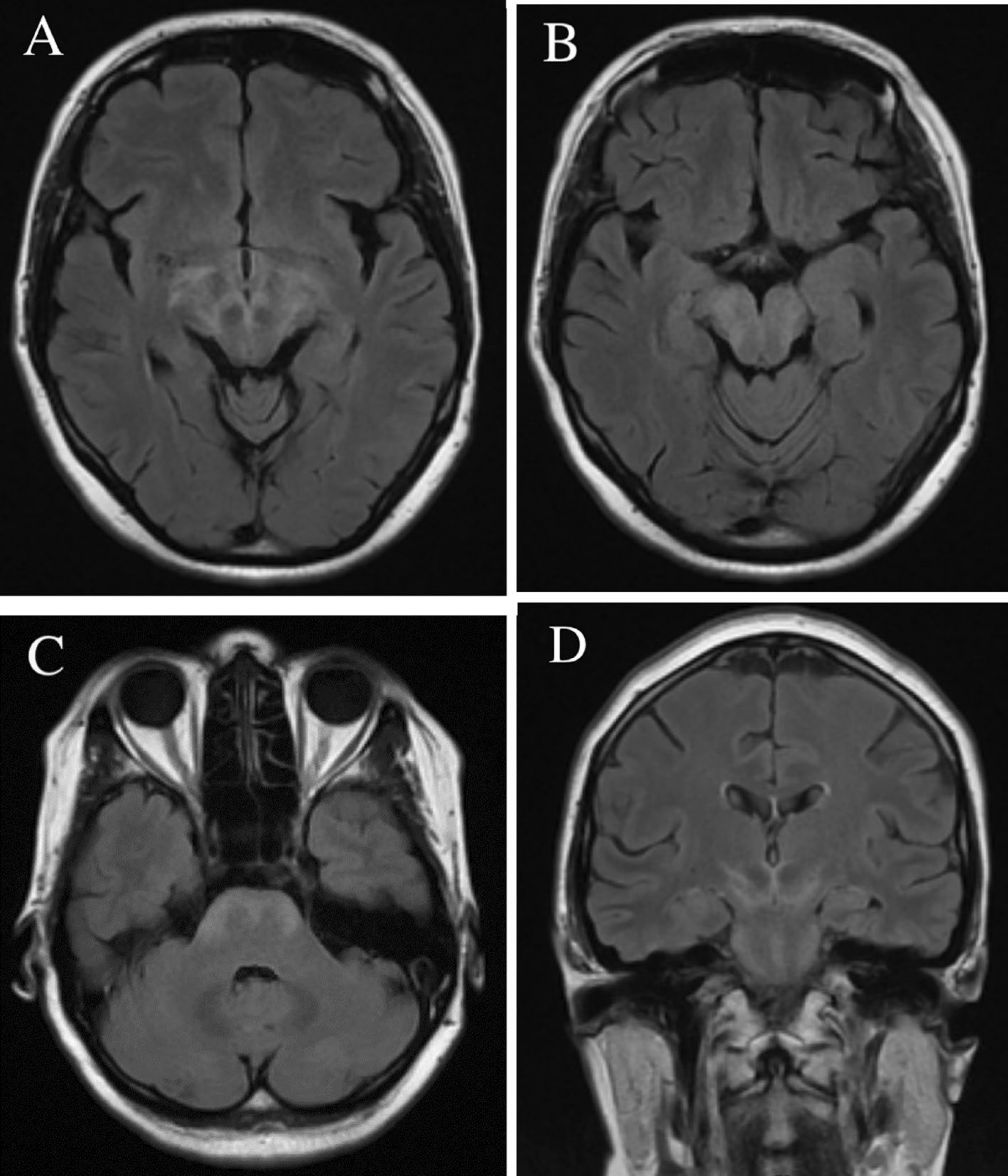
Head MRI of a patient diagnosed with Lyme neuroborreliosis (**A**–**C** axial T2 FLAIR, **D** coronal T2 FLAIR), showing confluent T2 hyperintense lesions involving basal ganglia, diencephalon, midbrain, and basal pons.

Normal values: protein (g/l) 0.15–0.45;

Head MRI was performed for 9/103 (8.7%) LNB patients, 5/9 (55.6%) scans were abnormal. The hyperintense T2 signal abnormalities, including small lesions in the subcortical and periventricular areas, deep white matter, and cerebellar hemispheres were found. One patient showed increased periventricular T2 signal. A case of confluent contrast-enhancing T2 lesions involving basal ganglia, diencephalon, midbrain, and pons is presented in Fig. 4. Cranial nerve contrast enhancement was prominent in four scans and involved oculomotor and facial nerves. Ten (9.7%) LNB patients underwent spinal MRI. Nine (90%) scans were abnormal. Two patients presenting myelitis had non-enhancing T2 hyperintense lesions in upper thoracic spinal cord, one lesion was segmental and involved posterior column, while the other scan showed transverse myelitis extending for three segments. Seven scans revealed miscellaneous pathology not involving nervous system (e.g. spondylosis or disc herniation). A detailed description of MRI findings is presented in supplementary Table A1 online.

Electrodiagnostic evaluation was performed for 9/103 (8.7%) patients with LNB. Nerve conduction studies (NCS) and/or electromyography (EMG) were performed to determine the severity of peripheral nervous system involvement. The clinically mild facial weakness was confirmed after doing facial nerve NCS with BLINK reflex in three cases. For other patients, NCS was performed for the evaluation of limb weakness. Time from the onset of the first muscle weakness signs to electrodiagnostic evaluation varied from 7 days to 6 months. All patients with limb weakness suffered severe radicular pains before the development of the paresis. For LNB patients with proximal limb weakness, the most common NCS finding was prolonged latencies of F waves – a sign of lesions in proximal parts or roots of the nerve. These findings and clinical features are summarized in supplementary Table A2 online.

### Treatment and outcomes

The median duration of hospitalization of LNB patients was 15.7 days ((IQR 10–19), min 1 − max 63). Patients with isolated polyradiculitis were treated in hospital for a shorter period compared to patients with facial palsy (13 (IQR 8.5–15) vs 16 (IQR 11–20), p = 0.014 respectively), or patients with encephalitis or myelitis (13 (IQR 8.5–15) vs 20 (IQR 18.5–28.75), p < 0.001 respectively) (Fig. 3). The median duration from the onset of symptoms to hospitalization was 20 days (Table 1). Patients with isolated peripheral facial palsy were hospitalized earlier compared to patients with polyradiculitis (median duration from symptom onset to hospitalization 5.5 days, IQR 1.5–7, min 0 − max 14, n = 10 vs 24 days, IQR 16–32.5, min 3 − max 63, n = 69, *p* < 0.001), as were patients with encephalitis (median 4 days, IQR 2–24, min 1 − max 33, n = 9, *p* = 0.006).

All patients with LNB were prescribed intravenous ceftriaxone. A total of seven LNB patients (6 with DNLB and 1 with PLNB) were treated with oral doxycycline 100 mg twice a day for 4–21 days before hospitalization, and all of them experienced the failure of treatment. Two of DNLB patients presented polyradiculitis. One patient presented polyradiculitis and peripheral facial palsy, one presented unilateral peripheral facial palsy, one developed bilateral peripheral facial palsy, and one patient developed polyradiculitis with oculomotor and abducens palsy. One PLNB patient presented polyradiculitis and developed peripheral facial palsy while taking doxycycline. All of these patients had good respond to intravenous ceftriaxone. The symptoms and signs of LNB progressed with intravenous ceftriaxone over a period of one week in three patients.

A total of 76/103 (73.8%) LNB patients were discharged with residual neurological symptoms or signs. The most common persisting neurological sign was unresolved peripheral facial palsy (40/103, 38.8%). Other unresolved neurological symptoms and signs were paresis (11/103, 10.7%), radicular pain (12/103, 11.7%), ataxia (3/103, 2.9%), ophthalmoplegia (1/103, 1%), and dizziness (1/103, 1%). One 88-year-old patient with encephalomyelitis died because of bacterial complications.

## Discussion

LNB has a variety of clinical manifestations. The results of our study showed that polyradiculitis, reported in 75% of cases, was the most common presentation of early LNB. This was also the first syndrome of LNB described by Garin, Bujadoux and Bannwarth^8^. Patients with Bannwarth syndrome have severe migrating radicular pain, which is not relieved by analgesics. The clinical picture of Bannwarth syndrome is often misinterpreted, and patients go through numerous investigations. In other studies, polyradiculitis was also the most common symptom with a frequency of 66%^9,10^ and 81%^11^. A total of 33% of our patients presented isolated polyradiculitis, which is similar to the study of Schwenkenbecher et al.^12^—25%, but lower than in the study of Nordberg et al.^13^—45%. Although polyradiculitis was the most common presentation and the most common initial symptom of early LNB, it was not the leading symptom for hospitalization. Patients with polyradiculitis had delayed hospitalization (median duration of symptoms before hospitalization 24 days vs. 5.5 days without polyradiculitis, *p* < 0.001). The developing peripheral facial palsy or encephalitic signs were the reasons for hospital admission. One of the most surprising findings was that as many as 91% of EM cases were untreated with antibiotics. It can be said that one third [31/100 (31%)] of early LNB cases could have been prevented if EM had been treated. Our results indicate that there is still a need for education of both professionals and the public about the most common symptoms of LB.

Peripheral facial palsy was the second leading manifestation of early LNB observed in 53% of cases in our study. In other studies, the rate of facial palsy was 52%^11^, 43%^12^, 41.3%^9^, and 36.4%^14^. According to our study, the rate of isolated facial palsy is 11%, while in the study of Schwenkenbecher et al.^12^ it was 16%. Univariable and multivariable regressions revealed that the absence of observed EM is associated with developing peripheral facial palsy. It is known that *B. afzelii* is associated with skin manifestation and *B. garinii* with nervous system involvement. It is not clear why patients without EM are more likely to develop facial neuropathy; microbiological studies are needed. It could be related to the characteristics of the causative agent—pathogenicity and tropism for certain cells of the nervous system. Univariable logistic regression revealed that age and male sex are predictors for developing peripheral facial palsy. Findings of Rojko et al.^15^ showed that age and delayed diagnosis are factors associated with the unfavorable outcome of peripheral facial palsy based on multiple regression analyses. A study by Petersen et al.^11^ reported a significantly lower risk of facial palsy without symptoms and signs of polyradiculitis in the group of patients aged 50 years and older, but study included only eight patients with facial palsy without radicular symptoms. In our study 11 patients with LNB had isolated facial palsy, and our findings closely paralleled those reported by Petersen et al.: we found a significantly lower risk of isolated facial palsy in the group of patients aged 50 years and older (OR 0.26, 95% CI 0.07–0.93, *p* = 0.038). However, it is important to note that the number of patients was limited, preventing us from drawing definitive conclusions.

It is very important for clinicians to know that patients with LNB may present symptoms and signs of encephalitis and myelitis. The rate of LNB encephalitis with or without myelitis was 13/103 cases (12.6%) in our study, similar to the German study^12^ (12%), but higher than in the Danish study^9^ (3.7%). LNB presenting as encephalitis is scarcely reported in literature^16^. The most common sign of encephalitis in our study was cerebellar ataxia (9/13 cases, 69.2%). Three patients (3/103, 2.9%) presented delirium, one (1/103, 0.97%) presented stupor, and two (2/103, 1.9%) presented dysphasia. In the study by Knudtzen et al.^16^, ataxia was reported in 11/45 cases (24.4%), confusion/disorientation in 20/45 cases (44.4%), and aphasia in 3/45 cases (6.7%). In literature, such rare signs of LNB encephalitis as impaired vision^17^, dementia-like syndromes^18,19^, and bilateral hearing loss^20^ are also reported.

We have found that patients with encephalitis and/or myelitis more often have a fever of ≥ 38 °C, and pleocytosis of ≥ 300 × 10^6^/l. This could be explained by a stronger inflammatory response in cases of encephalitis and/or myelitis compared to cases without the involvement of brain parenchyma. Astrocytes secrete inflammatory mediators following exposure to *B. burgdorferi* s.l*.* The studies also indicate that *Borrelia* can induce inflammatory responses in oligodendroglia and neuronal cells^8^. To find out more predictors and reasons for developing encephalitis and/or myelitis in patients with LNB, more studies are needed in endemic European countries. The results of our study showed that the rarest and most severe manifestation of early LNB is myelitis, which can result in death due to complications. Despite the rare occurrence, we, like other authors^16,21^, suggest that every patient presenting aseptic encephalitis or myelitis has to be tested for *B. burgdorferi* s.l. in endemic areas because unfavorable outcomes are possible.

It is known that *B. burgdorferi* s.l. does not usually cause any inflammatory changes in blood, but can rather trigger pronounced inflammatory changes in CSF. In this study, like in other studies, no abnormalities were found in routine blood laboratory parameters of LNB patients,^9,22^ but all patients had abnormalities in CSF. During LNB, *B. burgdorferi* s.l. invades the CSF. The host immune system reacts to the spirochete with inflammation^23^. LNB is characterized by B-cell activation, plasma cell infiltration, and enhanced intrathecal antibody production^8^. It is important to emphasize that the absence of *B. burgdorferi* s.l. antibodies in serum does not exclude early LNB. Increased level of *B. burgdorferi* s.l. antibodies was found in CSF but not in serum in 17% of cases in the study of Henningsson et al.^22^, and in 4% of cases in our study.

In the present study, seven patients presented neuroimaging lesions. Most commonly reported cerebral MRI findings were non-enhancing T2 lesions which were present in 15 to 63% of patients^24^ and were also present in more than a half of our patients’ scans. However, the specificity of such lesions is disputed by Agarwal et al.^25^, who did not observe the difference in incidence between LNB and control subjects. In our study, all but one patient with white matter T2 hyperintensities had a corresponding clinical syndrome. One patient without clinical signs of encephalitis had T2 hyperintense lesions in the cerebral hemispheres, another patient with pronounced ataxia had lesions in the cerebellar hemispheres, while the others with encephalitis had perivascular or subcortical white matter lesions. In addition, all these patients did not have history of vasculitis, heart disease, arterial hypertension, migraine or other conditions associated with nonspecific brain white matter hyperintensities. Nevertheless, given the high incidence in the general population, we can only state a possible relationship with LNB. Confluent contrast-enhancing lesions that were present in one of our patients are also reported in individual cases by others authors^25,26^. Other parenchymal manifestations of LNB include vasculitis^27^, hemorrhage^24^, mass lesions^28^, or hemorrhagic encephalitis^29^. Agarwal et al.^25^ reported three cases of nerve enhancement that corresponded to clinical signs of cranial neuritis. This was also observed in four of our patients. As in our study, meningeal enhancement is rarely reported in patients with LNB meningitis^5,25^. Lindland et al.^24^ found eleven case reports of LNB myelitis with associated MRI lesions—the majority showed longitudinal extensive and centrally or anteriorly located lesions in cervical spinal cord. Schwenkenbecher et al.^12^ reported MRI lesions located in cervical and thoracic segments in all five of their patients presenting with myelitis. We report two cases of spinal MRI lesions (transverse myelitis and segmental lesion of posterior column) located in upper thoracic segments. This might suggest that LNB myelitis does not have a strong predilection to a specific part of the spinal cord.

As many as 74% of LNB patients were discharged with unresolved symptoms or signs, the most common of which was unresolved peripheral facial palsy with a frequency of 39%. A total of 29% of patients had residual symptoms at discharge in the study of Krogen et al.^10^ Our results showed that peripheral facial palsy was not the most common initial symptom, as it was reported in only 14% of cases. The onset of the disease more commonly manifested as radicular pain, reported in 71% of cases, while peripheral facial palsy and other neurological deficits usually manifested later. Based on our data, we conclude that timely diagnosis and treatment of LNB are important to reduce the frequency of unfavorable outcomes.

In rare cases, patients can have coinfections, transmitted by ticks. All patients in our study were tested for tick-borne encephalitis (TBE), but none were tested for *B. miyamotoi. B. miyamotoi* disease, caused by spirochete of the relapsing fever group, is one of the emerging infectious diseases in Europe. Typically, patients experience fever with flu-like symptoms. Symptoms related to the central nervous system (CNS) can be observed^30^. We suggest testing all patients with fever and/or the inflammation in the CNS for *B. miyamotoi* in Lithuania and other countries where *Ixodes* ticks are prevalent.

This report comes with its own limitations: the study was carried out retrospectively, it was impossible to perform a statistical analysis of late LNB due to the small number of patients, and not all patients were monitored for residual effects after discharge from the hospital.

## Conclusion

The results of this retrospective study showed that polyradiculitis, reported in 75% of cases, was the most common neurological syndrome in patients with early LNB, and was also the most frequent first neurological sign but not the leading syndrome for hospitalization. Patients with polyradiculitis had delayed hospitalization. More than half of LNB patients developed peripheral facial palsy, and 14% of patients had encephalitis or myelitis. The absence of observed EM was the predictor of peripheral facial palsy, while female sex and EM untreated with antibiotics were predictors of isolated polyradiculitis. A fever of ≥ 38 °C and pleocytosis of ≥ 300 × 10^6^/l were associated with the development of encephalitis or myelitis in patients with early LNB.

## Methods

### Patients and study design

A retrospective study was performed to analyze the clinical and epidemiological features of LNB in hospitalized adults 18 years and older. The study took place in the Center of Infectious diseases and the Center of Neurology of Vilnius University Hospital Santaros Klinikos. These are referral centers for adult infectious diseases and neurology in eastern Lithuania. They serve a population of 809,000, which is 27% of the country’s population. The data of patients hospitalized in the years 2010–2021 were analyzed. Clinical and epidemiological data were collected from precisely documented medical records. The variables for the characterization of the cases were: age, sex, history of tick bite and EM observed by physician, co-morbidities, dates (of tick bite, EM, onset of symptoms and signs, hospitalization, discharge), clinical presentation, laboratory tests, instrumental findings, treatment, symptoms and signs on discharge.

DLNB was diagnosed if patients fulfilled the following three criteria: (1) had neurological symptoms suggestive of LNB (with other causes excluded); (2) had pleocytosis of ≥ 10 × 10^6^/l in CSF; (3) had intrathecally produced *B. burgdorferi* s.l. antibodies. PLNB was diagnosed if two out of these three criteria were fulfilled^31^. Other diseases such as TBE, syphilis, HIV infection, viral encephalitis (caused by HSV, VZV, HHV6, HHV7, enteroviruses, CMV, EBV), tuberculosis, sarcoidosis, multiple sclerosis and other demyelinating diseases, cancer, brain tumors, carcinomatous meningitis, oncological blood diseases were excluded. Early LNB was diagnosed if neurological symptoms and signs lasted for up to 6 months. Late LNB was diagnosed if neurological symptoms and signs presented for more than 6 months. Polyradiculitis was defined as the presentation of radicular pain with sensory impairment along at least two nerve roots, with or without radicular paresis. Patients presenting only symptoms and signs of polyradiculitis, concomitant with CSF pleocytosis of ≥ 10 × 10^6^/l, were classified as having isolated polyradiculitis. The pain was classified as radicular if it had following qualities: radiating nature, association with neuropathic phenomena (tingling, burning, etc.) or decreased sensation in the corresponding dermatomes or muscle weakness. Patients presenting only peripheral facial palsy, concomitant with CSF pleocytosis of ≥ 10 × 10^6^/l, were classified as having isolated peripheral facial palsy. Patients presenting symptoms and/or signs of brain damage such as focal neurological signs and/or impairment of consciousness, concomitant with CSF pleocytosis of ≥ 10 × 10^6^/l, were diagnosed with encephalitis. Patients with bilateral motor, sensory, or autonomic dysfunction attributable to spinal cord, concomitant with CSF pleocytosis of ≥ 10 × 10^6^/l, were diagnosed with myelitis. The diagnosis of EM was clinical: expanding red or bluish-red patch (≥ 5 cm in diameter), with or without center clearing^32^. Diagnosis of EM was made by physicians of the study centers. In cases where EM was not present during hospitalization diagnosis was based on the descriptions and photographs in the medical records.

### Laboratory testing

#### Calculation of AI

For the detection of intrathecal *B. burgdorferi s.l* antibodies production, CSF and peripheral blood samples were collected at the same time; IgG and IgM antibodies were analysed in one run at the Center of Laboratory medicine (Vilnius University Hospital Santaros Klinikos) using the quantitative immunoassay SERION ELISA classic *B. burgdorferi* IgG/IgM (Institut Virion/Serion GmbH, Wurzburg, Germany). Rheumatoid factors absorbent was used to pretreat samples before the IgM detection. To analyse dilution linearity of CSF and serum, the following combinations for dilutions of CSF and serum samples were used: CSF dilution 1:2 and 1:10, serum dilution 1:400 and 1:1000 (e.g., serum was diluted to CSF concentration). Concentrations of albumin, as well as total IgM and IgG levels in the CSF and serum were investigated by turbidimetric method using quantitative Optilite kits (The Binding Site Group Ltd., Birmingham, UK). In order to characterize the blood-CSF barrier functionality, as well as pathogen-specific antibodies production CSF/serum quotients of albumin, total and pathogen-specific IgM and IgG antibodies were calculated using SERION easy software, based on the scheme of prof. Hansotto Reiber. Borrelia-specific CSF/serum antibody index above 1.5 was considered to be pathological, indicative of intrathecal antibody synthesis directed against the pathogen, and supporting the diagnosis of LNB.

### Statistical methods

Continuous variables are presented as median [interquartile range (IQR)]. For categorical variables, absolute and relative frequencies were calculated. Mann–Whitney U test was used to compare continuous variables, and χ^2^ test or Fisher’s exact test was used to compare categorical variables. Univariable and multivariable binary logistic regression models were created to explore predictors associated with the development of isolated polyradiculitis, peripheral facial palsy, encephalitis and/or myelitis in patients with early LNB. Predictors were selected based on expert assessment. Odds ratio (OR) of 95% confidence interval (CI) was reported for logistic regression. *p* Value < 0.05 indicated statistical significance. IBM Statistical Package for the Social Sciences (SPSS) software version 20.0 was used for statistical analysis.

### Ethical statement

Vilnius Regional Biomedical Research Ethics Committee approved this study in 2022 (No. 2022/4-1426-897). The study was performed in accordance with the relevant guidelines and regulations. Informed consent was waived based on the Law on Biomedical Research of the Republic of Lithuania 2007, No. 125-5093 and the order of the director of the Lithuanian Bioethics Committee 2011, No. V-28.

### Supplementary Information

Supplementary Information.

## Data Availability

The datasets used and analyzed during this study are available from the corresponding author upon reasonable request.
