# Supplementary material for: Clinical and epidemiological features of Lyme neuroborreliosis in adults and factors associated with polyradiculitis, facial palsy and encephalitis or myelitis
Source: Sci Rep. 2023 Nov 14;13:19881. doi: 10.1038/s41598-023-47312-4 (PMC10646085; doi:10.1038/s41598-023-47312-4)
Supplement: Supplementary file 1 — Supplementary Information. [file 41598_2023_47312_MOESM1_ESM.docx]

**Clinical and epidemiological features of Lyme neuroborreliosis in adults and factors associated with polyradiculitis, facial palsy and encephalitis or myelitis**

Daiva Radzišauskienė^1*^, Jurgita Urbonienė^2^, Arminas Jasionis^3^, Aušra Klimašauskienė^3^, Radvilė Malickaitė^4^, Agnė Petrulionienė^5^, Monika Vitkauskaitė^2^ & Gintaras Kaubrys^3^

^1^Clinic of Infectious Diseases and Dermatovenerology, Institute of Clinical Medicine, Faculty of Medicine, Vilnius University, Lithuania

^2^Center of Infectious Diseases, Vilnius University, Lithuania

^3^Clinic of Neurology and Neurosurgery, Institute of Clinical Medicine, Faculty of Medicine, Vilnius University, Lithuania

^4^Clinic of Cardiac and Vascular Diseases, Institute of Clinical Medicine, Faculty of Medicine, Vilnius University, Lithuania

^5^Constitution Clinic, Vilnius, Lithuania

^*^Corresponding author

E-mail address: [daiva730jvg@gmail.com](mailto:daiva730jvg@gmail.com)

Table A1. Detailed radiological findings in MRT related to Lyme neuroborreliosis

| Patient ID | Radiological finding | Clinical syndromes | Pathological CSF/serum *B. burgdorferi* s.l. IgM AI | Pathological CSF/serum *B. burgdorferi* s.l. IgG AI |
| --- | --- | --- | --- | --- |
| 45 | T2 hyperintense lesions in cerebellar hemispheres without CE | Peripheral facial palsy;  Encephalitis with ataxia;  Polyradiculitis | _ | + |
| 96 | Increased periventricular T2 signal, without CE;  CE in intracranial parts of facial nerve | Encephalitis;  Peripheral facial palsy;  Polyradiculitis with leg paresis | + | _ |
| 110 | Confluent T2 hyperintense lesions extending in basal ganglia, midbrain, pons and medulla oblongata with CE in oculomotor and facial nerve | Encephalitis;  Ophthalmoplegia, facial numbness;  Polyradiculitis | + | + |
| 127 | T2 hyperintense lesions in cerebral hemispheres (subcortical, periventricular);  CE of oculomotor and facial nerve | Peripheral facial palsy;  Ophthalmoplegia | + | + |
| 159 | T2 hyperintense lesions in deep white matter without CE | Encephalitis;  Polyradiculitis | + | + |
| 23 | Segmental T2 hyperintense lesion at Th1-3 level (posterior column) | Encephalomyelitis | _ | + |
| 50 | Transverse T2 hyperintense lesion at Th1-3, level | Encephalomyelitis | _ | + |

AI – antibody index, CE – contrast enhancement, CSF – cerebrospinal fluid

Table A2. Clinical characteristics and electrodiagnostic findings of patients with Lyme neuroborreliosis

| Patient | Sex, age | Time of evaluation from first symptoms | Clinical features | ENMG findings |
| --- | --- | --- | --- | --- |
| 1 | Female, 77 | 6 months | Left facial weakness, asymmetric tetraparesis with more affected distal parts, only left leg weakness and limb paresthesias in the beginning.  Slow progression despite adequate treatment during the 6 months. | Tested nerves: median and ulnar (motor and sensory) with F waves, tibial and peroneal with F waves, sural bilaterally.  Results:  No responses of hand nerves on both sides;  Normal sural responses;  No motor nerves responses on the right leg, left peroneal nerve response of low amplitude, latency and conduction velocity normal, tibial nerve response and F waves latencies within normal range;  EMG of leg muscles: spontaneous activity (fibrillations, positive sharp waves, few fasciculations), hips muscles motor unit action potentials of prolonged duration and amplitude, no motor unit action potentials in calf muscles;  Arms muscles: spontaneous activity (fibrillations, positive sharp waves, few fasciculations), motor unit action potentials in proximal arm muscles of prolonged duration and amplitude;  Conclusion of ENMG: findings are compatible with the diagnosis of severe asymmetric axonal polyradiculoneuropathy, denervation signs with ongoing reinnervation in proximal muscles are present. |
| 2 | Male, 40 | 6 weeks | Mild weakness of the left leg, the left facial weakness. | Tested nerves: tibial and peroneal with F waves, sural bilaterally;  Results: data within normal values;  EMG of tibial anterior muscle (both sides) – only a few fasciculations; vastus medial and gastrocnemius muscles – normal data. |
| 3 | Male, 87 | 4 weeks | Bilateral asymmetric proximal arm weakness. | Tested nerves: median and ulnar (sensory and motor) with F waves, axillar, n. musculocutaneous;  Results: diminished amplitude of axillar nerve response, prolonged median F waves;  EMG: signs of acute denervation – spontaneous activity (fibrillations and fasciculations) without signs of reinnervation bilateral in deltoid, biceps and triceps muscles;  Conclusion: axonal damage of proximal nerves bilaterally, sings of axonal loss. |
| 4 | Female, 71 | 4 weeks | Proximal leg weakness. | Tested nerves: peroneal, tibial with F waves and sural bilaterally, median and ulnar, sensory and motor with F waves on the right side;  Arm nerves – normal values;  Sural responses absent (finding of undetermined significance because of age of patient);  Tibial and peroneal amplitudes at lower limit of normal, F waves of tibial nerves prolonged bilaterally ;  Conclusion: suspected proximal motor nerves damage. |
| 5 | Female, 57 | 7 days | Left facial and proximal right leg weakness. | Tested nerves: median and ulnar, motor and sensory with F waves; tibial and peroneal with F waves and sural bilaterally; facial nerves with BLINK reflex bilaterally  Results: limb nerves – normal values, peripheral damage of left facial nerve |
| 6 | Female, 61 | 2 months | Severe radicular pain with doubtful proximal leg weakness | Tested nerves: tibial and peroneal with F waves and sural bilaterally;  Results: normal tibial and peroneal responses, F waves of prolonged latencies;  Conclusion: possible proximal motor nerves involvement. |
